# The added value of chemotherapy after secondary cytoreductive surgery in unifocal versus multifocal first recurrent epithelial ovarian cancer: A systematic review^☆^

**DOI:** 10.1016/j.gore.2025.101809

**Published:** 2025-07-18

**Authors:** R.E.W.M. van de Vorst, M.D. Huiskamp, E.H. Gort, P.O. Witteveen, R.P. Zweemer, C.G. Gerestein

**Affiliations:** a Department of Gynecological Oncology, Division of Imaging and Oncology, University Medical Center Utrecht, Utrecht, the Netherlands; b Department of Medical Oncology, Division of Imaging and Oncology, University Medical Center Utrecht, Utrecht, the Netherlands

## Abstract

**Objective:**

This systematic review aims to evaluate the added value of chemotherapy after secondary cytoreductive surgery (SCS) on survival in patients with unifocal and multifocal first recurrent epithelial ovarian cancer (EOC). Moreover, it compares survival outcomes between unifocal and multifocal recurrences in treatment outcomes.

**Methods:**

A systematic search was conducted across PubMed, Embase, and the Cochrane Library identified 907 articles. Studies were selected if they involved patients with first recurrent EOC undergoing SCS, stratified by unifocal or multifocal recurrence. The primary outcomes were progression-free survival (PFS) and overall survival (OS).

**Results:**

No studies specifically addressed the added value of chemotherapy after SCS in unifocal versus multifocal first recurrent EOC. This systematic review identified eight studies examining PFS and OS in patients with unifocal and multifocal first recurrent EOC following SCS with chemotherapy. Findings consistently show that unifocal first recurrent EOC is associated with significantly improved PFS and OS compared to multifocal first recurrent EOC. However, the extent of the survival benefit varied. Six studies showed an advantage for unifocal recurrence on multivariate analyses, although two studies did not find statistically significant differences after adjusting for other variables.

**Conclusion:**

This review shows that there is a knowledge gap regarding the added value of chemotherapy after complete SCS in first recurrent EOC. However, this review shows a survival advantage of unifocal over multifocal first recurrent EOC. There is a need for clinical trials to compare survival outcomes between unifocal and multifocal first recurrent EOC patients undergoing SCS alone or SCS with chemotherapy.

## Highlights

• No studies assess chemotherapy after secondary debulking in unifocal versus multifocal first recurrent ovarian cancer.
• Unifocal first recurrent EOC shows better PFS and OS than multifocal recurrence after secondary debulking with chemotherapy.
• Future RCTs should evaluate the added value of chemotherapy after secondary debulking in unifocal first recurrent EOC.

## 1 Introduction

After completion of primary treatment patients with advanced epithelial ovarian cancer (EOC) have an 80% chance of recurrence, which mostly presents as multifocal disease (Armstrong et al., 2006). In 30 to 40% of the patients recurrence is unifocal (Oksefjell, 2011). Recurrent disease is generally treated with chemotherapy alone or secondary cytoreductive surgery (SCS) followed by chemotherapy in a selected group of patients.

The DESKTOP-III and SOC-1 randomized controlled trials have recently investigated the impact of SCS on survival outcomes. Patients with a platinum-sensitive first EOC recurrence were randomized to receive either chemotherapy alone or SCS followed by chemotherapy.

The previous DESKTOP-I and DESKTOP-II trials have identified three prognostic parameters of achieving complete resection during SCS, collectively referred to as the Arbeitsgemeinschaft Gynäkologische Onkologie (AGO) score. A positive AGO score is defined by an Eastern Cooperative Oncology Group (ECOG) performance status of 0, complete resection during primary debulking, and the absence of ascites (<500 ml). The DESKTOP-III trial included 407 patients based on a positive AGO score and the estimation of an experienced gynecological oncologist if complete tumor resection is feasible (Harter, 2021). The trial found a significant prolongation of median progression-free survival (PFS) by 4.4 months and median overall survival (OS) by 7.7 months for patients who were treated with SCS and chemotherapy (PFS 18.4 months; OS 53.7 months) compared to chemotherapy alone (PFS 14.0 months; OS 46.0 months). Both patients with unifocal and multifocal recurrences were included in this trial, however, no survival analysis has been performed for these subgroups.

The SOC-1 trial included 357 patients based on the prediction that complete tumor resection was deemed feasible using the international iMODEL-score and PET-CT imaging (Shi et al., 2021). The trial demonstrated a significant prolongation of median PFS by 6.1 months for patients treated with SCS and chemotherapy (PFS 18.0 months) compared to chemotherapy alone (PFS 11.9 months). In the intention-to-treat population, the median OS for the SCS and chemotherapy group (58.1 months) exceeded that of the chemotherapy group (52.1 months) by 6.0 months, although this difference was not statistically significant. After adjusting for crossover, the median OS in the control group was determined to be 49.5 months (Jiang et al., 2024). The adjusted hazard ratio for dead following crossover to surgery was 0.76 (95 % CI, 0.58 to 0.99), indicating a significant risk reduction associated with SCS and chemotherapy. Moreover, a prespecified subgroup analysis by tumor lesions showed that patients with 1–3 lesions (102.6 months) had the most favorable OS, followed by 4–19 lesions (69.5 months) and ≥ 20 lesions (36.3 months). Patients with 1–3 lesions had a significant survival advantage in comparison to patients with ≥20 lesions (HR 0.39, 95 % CI: 0.28–0.55).

Patients in whom complete tumor resection was achieved experienced the most pronounced OS benefits, as demonstrated by OS benefits of 61.9 months and 73 months, in the DESKTOP-III and SOC-1 trials respectively. These studies show the importance of SCS, which aligns with the importance of complete debulking in the primary EOC setting. Higher proportions of complete resection after SCS are observed in patients with unifocal recurrences referred to as recurrence at a single intra-abdominal lesion (Munkarah et al., 2001); (Zang et al., 2004) . Moreover, in a retrospective review patients with a first platinum-sensitive unifocal recurrence at one tumor lesion, is independently associated with complete resection (Gronlund et al., 2005). However, the added value of chemotherapy after SCS was not questioned in these trials.

This systematic review aims to evaluate the added value of chemotherapy after SCS for first recurrence of EOC and identify the prognostic differences in treatment outcomes between unifocal and multifocal first recurrent EOC which was not addressed in the studies mentioned above.

## 2 Methods

### 2.1 Systematic search

This systematic review was conducted in accordance with the PRISMA (Preferred Reporting Items for Systematic Reviews and Meta-Analysis) guidelines (Appendix Table A1) (Page et al., 2021). A systematic search was performed across the Pubmed (MEDLINE), Embase, and the Cochrane Library databases on 15–11-2024. The following search terms were used: “ovarian cancer”, “recurrence”, “cytoreductive surgery”, “progression-free survival”, and “overall survival”, along with their synonyms and alternative spellings. Additional literature was obtained by cross-examining the references of the retrieved articles.

### 2.2 Selection and eligibility

Articles identified were screened for eligibility and duplicate removal by M.H., based on titles and abstracts, using Rayyan software (Qatar Computing Research Institute, HBKU, Doha, Qatar) (H. H. Z. F. and A. Elmagarmid. Mourad Ouzzani, ‘Rayyan — a web and mobile app for systematic reviews’, 2016). A second reviewer, R.V., verified study eligibility, with any discrepancies resolved through consensus discussions. Studies were included in our systematic review if they met the following predefined criteria: (1) randomized controlled trials, case reports, prospective or retrospective cohort studies, (2) patients with a first recurrent EOC treated with SCS, (3) unifocal recurrence, including all synonyms, refers to recurrence at one or two tumor lesions and multifocal recurrence refers to as more than two tumor lesions, (4) PFS and (5) OS.

Studies were excluded when they were: (1) non-English, non-human studies or if no full-text article was not available, (2) performed hyperthermic intraperitoneal chemotherapy during surgery, and (3) original data was not reported.

### 2.3 Quality assessment

The Newcastle-Ottawa Scale (NOS) was applied by two independent reviewers, R.V. and M.H., to systematically assess the quality of all eligible articles and collect data from all included studies. Any discrepancies were resolved through discussion and consensus between reviewers. The NOS evaluates study quality across three domains: selection, comparability, and outcome. Studies could achieve a maximum score of 9 points, which reflects the highest quality for a well-designed, double-arm cohort study. Studies with an overall score of 6 or lower, indicating a high risk of bias, were excluded from the review.

### 2.4 Study outcomes

The primary outcome of this systematic review was to compare PFS and OS in patients with first recurrent EOC treated with SCS alone versus SCS followed by chemotherapy considering unifocal and multifocal recurrence. Second, we examined PFS and OS differences in treatment outcomes between unifocal and multifocal first recurrent EOC.

## 3 Results

The primary aim of this systematic review to compare PFS and OS in patients with unifocal versus multifocal first recurrent EOC treated with either SCS alone or SCS followed by chemotherapy yielded no eligible articles. This question could not be answered based on the current available literature. Our secondary aim was to compare PFS and OS of patients with unifocal and multifocal first recurrent EOC undergoing SCS with chemotherapy.

### 3.1 Systematic search and quality analysis

**Fig. 1:**
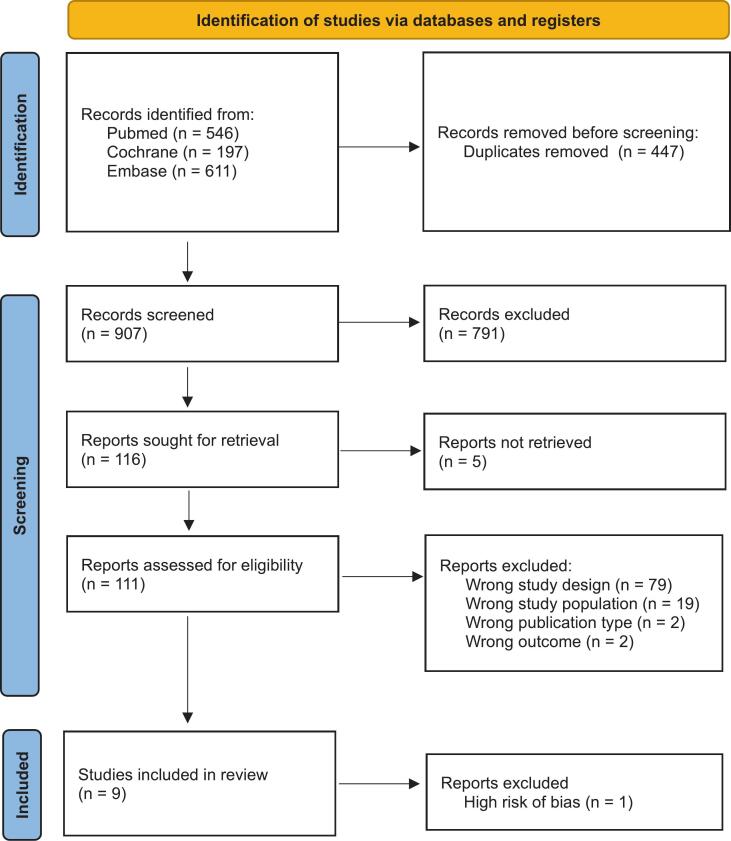
PRISMA flowchart of the systematic search**.** .

**Source: Page MJ, et al. BMJ 2021;372:n71. 10.1136/bmj.n71.This work is licensed under CC BY 4.0. To view a copy of this license, visit****https://creativecommons.org/licenses/by/4.0/**

The systematic search resulted in 907 unique articles. Following title-abstract screening and full-text assessment, 9 articles met the inclusion criteria (Fig. 1). Quality assessment resulted in the exclusion of one article due to a high risk of bias (Appendix Table A2). Primary quality issues identified in the included studies were the absence of a control group and inadequate reports of a number of patients due to a loss of follow-up.

### 3.2 Progression-free survival and overall survival

Eight retrospective cohort studies reported PFS and/or OS in patients with unifocal and multifocal first recurrence of EOC following SCS with chemotherapy (Table 1) (Boran, 2012, Fan et al., 2017, Oksefjell et al., 2009, Onda et al., 2005, Salani, 2007, So, 2019, Ye et al., 2020, Chi, et al., 2006). The median reported follow-up duration ranged from 36.9 to 60 months (Boran, 2012, Fan et al., 2017, Oksefjell et al., 2009, Onda et al., 2005, Salani, 2007, So, 2019, Ye et al., 2020, Chi, et al., 2006). In most studies, unifocal recurrence was defined as a single lesion and multifocal as more than one. Boran et al. and Oksefjell et al. deviated by including up to two lesions as unifocal (Boran, 2012, Oksefjell et al., 2009). Salani et al. defined multifocal disease as three to five lesions (Salani et al., 2007). Chi et al. introduced a separate category for carcinomatosis (≥20 lesions), which was excluded from the multifocal group (Chi, et al., 2006).

**Table 1:** Progression-free survival and overall survival for unifocal and multifocal first recurrence of epithelial ovarian carcinoma following secondary cytoreductive surgery and chemotherapy.

Study		Unifocal (months)	Multifocal (months)	P-value
Boran et al. (2012)	n	31	36	
	PFS	15	1	0.002
	OS	25	17	
Chi et al. (2005)	n	41	68	
	PFS	−	−	
	OS	60.3	41.7	<0.001
Fan et al. (2016)	n	28	75	
	PFS	48	30	0.0219
	OS	71	52	0.0308
Oksefjell et al (2008)	n	84	133	
	PFS	−	−	
	OS	40.8	12	<0.001
Onda et al. (2005)	n	16	28	
	PFS	−	−	
	OS	64	27	0.0070
Salani et al. (2007)	n	35	20	
	PFS	−	−	
	OS	52	12	<0.001
So et al. (2019)	n	6	16	
	PFS	−	−	
	OS	60.2	13.5	<0.044
Ye et al. (2020)	n	29	16	
	PFS	19	11	0.0230
	OS	−	−	0.0290

Chi et al. included 153 patients, of whom 129 received platinum-based chemotherapy, 21 non-platinum-based treatment and 3 with unknown additional treatment (Chi, et al., 2006). Ye et al. included 45 patients with recurrent ovarian clear cell carcinoma subtype who underwent SCS, with an unspecified number receiving chemotherapy (Ye et al., 2020). Salani et al. included 55 patients receiving SCS with chemotherapy, primarily stratifying on the number of lesions (Salani et al., 2007).

Chi et al. reported a significant different median OS among unifocal recurrence, multifocal recurrence, and for a separate group with carcinomatosis (Chi, et al., 2006). Oksefjell et al. found a significantly prolonged median OS for unifocal recurrence compared to multifocal recurrence and cases where surgery was not performed (Oksefjell et al., 2009). Ye at al. found significant differences in OS favoring unifocal recurrence, although precise survival data were not disclosed (Ye et al., 2020).

The findings of the included studies consistently indicate that a unifocal first recurrence of EOC following SCS was associated with significantly improved PFS and OS compared to multifocal first recurrence (Boran, 2012, Fan et al., 2017, Oksefjell et al., 2009, Onda et al., 2005, Salani, 2007, So, 2019, Ye et al., 2020, Chi, et al., 2006). While the majority of studies reported longer median PFS and OS for unifocal recurrence, the extent of the survival benefit varied across studies. Multivariate analyses for PFS and OS showed in six studies a survival advantage for unifocal recurrence, although Salani et al. and So et al. did not find statistically significant differences after adjusting for other variables (Table 2, Table 3) (Salani, 2007, So, 2019). Salani et al. observed a drop to borderline significance (p = 0.06) for number of recurrence lesions when adjusted for disease-free interval, number of recurrence lesions at imaging and complete cytoreduction after SCS (Salani et al., 2007). When adjusted for age, the significance of complete cytoreduction after primary debulking and performance status at SCS, by So et al. dropped to a p-value of 0.075 when comparing unifocal versus multifocal first EOC recurrences (So et al., 2019).

**Table 2:** Multivariate analysis for progression-free survival in unifocal and multifocal first recurrence of epithelial ovarian carcinoma following secondary cytoreductive surgery and chemotherapy.

Study	Measurement of association	Mean	Lower limit	Upper limit	P-value
Boran et al. (2012)	OR	2.334	1.329	4.097	0.003
Fan et al. (2016)	HR	1.409	1.028	2.545	0.027
Ye et al. (2020)	HR	1.826	0.695	4.797	0.222

**Table 3:** Multivariate analysis for overall survival in unifocal and multifocal first recurrence of epithelial ovarian carcinoma following secondary cytoreductive surgery and chemotherapy.

Study	Measurement of association	Mean	Lower limit	Upper limit	P-value
Fan et al. (2016)	HR	1.514	1.122	2.773	0.032
Onda et al. (2005)	RR	3.730	1.790	9.580	<0.001
So et al. (2019)	HR	4.242	0.865	20.82	0.075
Ye et al. (2020)	HR	4.059	1.684	9.784	0.002

## 4 Discussion

This systematic review shows a significant knowlegde gap regarding survival outcomes following secondary cytoreductive surgery (SCS) alone versus SCS followed by chemotherapy in patients with unifocal and multifocal first recurrent epithelial ovarian cancer (EOC). No eligible studies specifically addressing this question were identified. This highlights the need for future research to gather insight into survival outcomes of SCS alone in this specific patient population.

This review finds across the eight included studies that unifocal first EOC recurrence is associated with significantly improved progression-free survival (PFS) and overall survival (OS) compared to multifocal first EOC recurrence after SCS with chemotherapy (Boran, 2012, Fan et al., 2017, Oksefjell et al., 2009, Onda et al., 2005, Salani, 2007, So, 2019, Ye et al., 2020, Chi, et al., 2006). We hypothesize that this survival advantage might be attributed to the higher likelihood of achieving complete tumor resection in unifocal recurrences. The DESKTOP OVAR trial identified complete resection as the only prognostic factor for prolonged overall survival in recurrent EOC, (median OS 45.2 vs. 19.7 months; HR 3.71; 95 % CI 2.27–6.05; P < 0.0001), while the AGO-OVAR trial confirmed its importance in primary debulking (Du Bois et al., 2009, Harter, 2006). Multivariate analyses in six studies upheld statistical significance for the survival advantage in unifocal recurrence. However, Salani et al. and So et al. did not find these differences to remain significant after adjusting for potential confounders, which included disease-free interval (DFI), the number of recurrence sites observed on imaging, performance status at SCS, and complete cytoreduction after primary debulking and SCS. These factors may influence survival outcomes (Salani, 2007, So, 2019).

Eligibility for SCS in recurrent EOC was uniformly based on the DFI, tumor resectability, and patient’s performance status. Additional criteria included age, used by Oksefjell et al. and Onda et al., and the absence of ascites, emphasized by Boran et al. and So et al. (Boran, 2012, Oksefjell et al., 2009, Onda et al., 2005). The Tian model, employed by So et al., further integrated CA-125 levels, initial tumor stage, and residual disease (So et al., 2019). Most studies required a DFI of at least six months, reflecting platinum-sensitive recurrence. Fan et al. introduced a higher threshold of 12 months, suggesting that a longer DFI correlates with more favourable prognostic outcomes (Fan et al., 2017). Ye et al. uniquely focused on patients with the clear cell subtype of EOC, contrasting with other studies that included all histological subtypes (Ye et al., 2020).

Classification based on disease extent varied across studies. While most defined unifocal recurrence as a single lesion and multifocal as more than one lesion, Boran et al. and Oksefjell et al. considered up to two lesions as unifocal, and Salani et al. categorized three to five lesions as multifocal (Boran, 2012, Oksefjell et al., 2009, Salani, 2007). Chi et al. introduced a subgroup for patients with carcinomatosis, characterized by 20 or more lesions, excluding these from the multifocal recurrence subgroup (Chi, et al., 2006). These inconsistencies may influence resectability assessments and subsequent prognoses. Patients categorized as unifocal in some studies may have had a disease burden comparable to multifocal cases in other studies, affecting reported resectability rates. Similarly, patients with carcinomatosis have a higher likelihood of incomplete tumor resection. Excluding these patients from the multifocal group could overestimate its prognosis and contribute to heterogeneity in study outcomes. Notably, none of the included studies excluded patients with incomplete resection from either the unifocal or multifocal groups. We should aim for an universally accepted threshold to distinguish between unifocal and multifocal recurrence. Classifications incorporating methods like the peritoneal carcinomatosis index (PCI), as utilized in primary EOC, can standardize assessments of disease extent and enhance consistency in treatment decisions.

Surgery combined with subsequent platinum-based chemotherapy was the dominant treatment approach. Onda et al. uniquely included patients treated with chemotherapy prior to surgery, provided no progression occurred during the preoperative chemotherapy phase (Onda et al., 2005). Definitions of OS and PFS were generally consistent, with OS measured from surgery to death and PFS from surgery to the second relapse. Fan et al. measured OS and PFS from diagnosis instead of surgery, a distinction unlikely to have clinical relevance, as treatment typically begins promptly after diagnosis (Fan et al., 2017).

Building on previous research, Lee et al. examined the impact of SCS on survival in patients with platinum-sensitive recurrent ovarian cancer in the CALYPSO trial (Lee et al., 2015). Their findings revealed a significant hazard ratio (HR) of 1.28 (95 % CI: 1.06–1.54, *p =* 0.01) for PFS and OS when the number of metastatic organ sites was dichotomized into one lesion versus more than one lesion in multivariate analysis. Similarly, Zhao et al. investigated the added value of SCS in patients with recurrent ovarian cancer (Zhao et al., 2024). They reported that the extent of the disease, classified as single versus multiple lesions, was significantly associated with reduced PFS (HR 4.652; 95 % CI: 1.273–17.04, *p =* 0.020). However, this association could not be established for OS (HR 1.444; 95 % CI: 0.878–2.374, *p =* 0.148).

Given the superior outcomes associated with unifocal recurrence, this review raises the question of complete resection rates in unifocal and multifocal recurrences and whether chemotherapy provides additional benefit in patients with completely resected unifocal first recurrent EOC. In the absence of measurable disease the benefit of chemotherapy is difficult to assess. Overtreatment with chemotherapy may induce platinum resistance, limiting future treatment options and diminishing eligibility for therapies like PARP inhibitors. Delaying chemotherapy until a second recurrence may preserve platinum sensitivity, balancing immediate treatment needs with long-term therapeutic prospects.

Although the clinical implications of platinum-based combination chemotherapy in ovarian cancer patients are well-established, it is important to recognize that this treatment also carries significant risks of complications (Parmar et al., 2003). Platinum-based chemotherapy is associated with significant toxicities in up to 95 % of the patients, including nephrotoxicity, neurotoxicity, ototoxicity, and severe nausea, which can adversely affect quality of life (Butler et al., 2004). These risks must be weighed against the uncertain survival benefits of chemotherapy in patients with unifocal recurrence, particularly when complete resection is achieved. Future research is needed to address the added value of chemotherapy after SCS in unifocal first recurrent EOC.

The key message of this systematic review is its distinction between unifocal and multifocal first EOC recurrences and their respective impact on survival outcomes after SCS with chemotherapy. This systematic review has certain limitations. All included studies were retrospective cohort studies, which inherently carry risks of selection bias and confounding. Additionally, the studies exhibited considerable heterogeneity in study design, patient populations, and outcome reporting, precluding the feasibility of performing a meta-analysis. We attempted to control these issues by applying strict inclusion criteria and rigorous quality assessment to ensure that only the most reliable studies were included.

## 5 Conclusions

This systematic review showed the lack of evidence on the added value of chemotherapy after SCS on survival outcomes in patients with first recurrent EOC with a special focus on unifocal versus multifocal recurrence. Clinical studies are needed to address this issue. The review did reveal that unifocal recurrences were consistently associated with improved PFS and OS compared to multifocal first recurrent EOC after SCS with chemotherapy. Variations in eligibility criteria, recurrence classifications, and treatment regimens complicate direct comparisons between studies.

Given the uncertainty surrounding the added value of chemotherapy after SCS in patients with completely resectable unifocal recurrences and its potential toxicity, future research is essential.

## Ethical approval

Not applicable.

## Author contribution

The research question was formulated by RV, MH, PW, RZ and CG. Methods were designed by RV, MH, PW, RZ and CG. The search, risk of bias assessment, and data collection were performed by RV and MH. Results were interpreted by all authors. The manuscript was drafted by RV and MH and critically revised by EG, PW, RZ, and CG.

## CRediT authorship contribution statement

**R.E.W.M. van de Vorst:** Writing – original draft, Methodology, Conceptualization. **M.D. Huiskamp:** Methodology, Conceptualization. **E.H. Gort:** Writing – review & editing. **P.O. Witteveen:** Writing – review & editing, Supervision, Methodology, Conceptualization. **R.P. Zweemer:** Writing – review & editing, Supervision, Methodology, Conceptualization. **C.G. Gerestein:** Writing – review & editing, Supervision, Methodology, Conceptualization.

## Funding

This research did not receive any specific grant from funding agencies in the public, commercial, or not-for-profit sectors.

## Declaration of competing interest

The authors declare that they have no known competing financial interests or personal relationships that could have appeared to influence the work reported in this paper.
